# Incidental Finding of a Retained Foreign Body Following Unintentional Gunshot Injury

**DOI:** 10.7759/cureus.96061

**Published:** 2025-11-04

**Authors:** Muhammad Ahmad Majid, Mohammad J Faisal, Muhammad Najam Us Saqib, Mahnoor Shahid, Muhammad Awais Waheed

**Affiliations:** 1 Department of Surgery, Fauji Foundation Hospital (FFH), Rawalpindi, PAK; 2 Department of Surgery, Cambridge University Hospitals, Cambridge, GBR; 3 Department of Surgery, Foundation University Medical College, Islamabad, PAK; 4 Department of Internal Medicine, Fauji Foundation Hospital (FFH), Rawalpindi, PAK

**Keywords:** asymptomatic, firearm injury, general surgery, gunshot wound, incidental radiological finding, retained foreign object, x-ray

## Abstract

Firearm injuries are increasing worldwide and contribute significantly to morbidity and mortality. While most cases present with obvious trauma in emergency departments, there are rare instances of gunshot injuries that manifest with atypical signs and symptoms, offering minimal indication of a firearm wound. We report a similar case of a child who presented with a minor wound on the forearm without any history of trauma or other identifiable cause, which was later revealed on X-ray to be a gunshot wound with a retained bullet in the right forearm. The patient was asymptomatic. The bullet was surgically removed under general anesthesia without complications. The patient recovered uneventfully and was discharged the next day, with a follow-up scheduled in two weeks for suture removal. This report aims to highlight the importance of imaging in seemingly trivial injuries of unknown cause and the need for judicious decision-making when considering interventional management options.

## Introduction

Gunshot wounds (GSWs) remain a major public health issue, especially among young males in low- and middle-income countries [1,2]. The majority of such injuries present with obvious trauma, bleeding, bone fractures, and the typical signs of a GSW. However, a subset of cases may present atypically, with minimal external signs and no known history of firearm exposure. In such cases, retained bullets may be discovered incidentally, posing diagnostic and management challenges [3].

In regions like South Asia, celebratory aerial firing is a culturally rooted yet dangerous practice. According to a report published in Dawn News on August 15, 2023, at least three people were killed and 119 others were injured in incidents of aerial firing across Karachi, Pakistan, during Independence Day celebrations [4]. Stray or falling bullets fired into the air may descend at terminal velocity and cause injuries, often without an audible gunshot. In some cases, such injuries may go unrecognized until imaging is performed [2,5,6].

The management of asymptomatic retained bullets remains debated. While conservative treatment, including no intervention with regular follow-up for monitoring, is often preferred in asymptomatic cases, surgical removal is considered when there is a risk of neurovascular injury, infection, migration, or lead toxicity. Each case is therefore managed individually, considering the risks of treatment and the potential complications that may arise if left untreated [3,7,8].

We present the case of a 12-year-old male from Pakistan who presented with a seemingly minor forearm wound without a history of trauma and was found to have a retained bullet, highlighting the importance of imaging techniques even in small, asymptomatic wounds with minimal suspicion of a foreign body.

## Case presentation

A 12-year-old male from Rawalpindi, Pakistan, presented to the ED on February 16, 2024, with a single wound on the dorsal aspect of his right forearm. He reported experiencing sudden, sharp pain while standing on the roof of his house and inspecting the rooftop water tank about an hour earlier. He noticed a puncture-like wound with mild bleeding but denied any known trauma and could not identify the cause of the injury. He was otherwise healthy and had no significant prior medical or surgical history.

**Figure 1:**
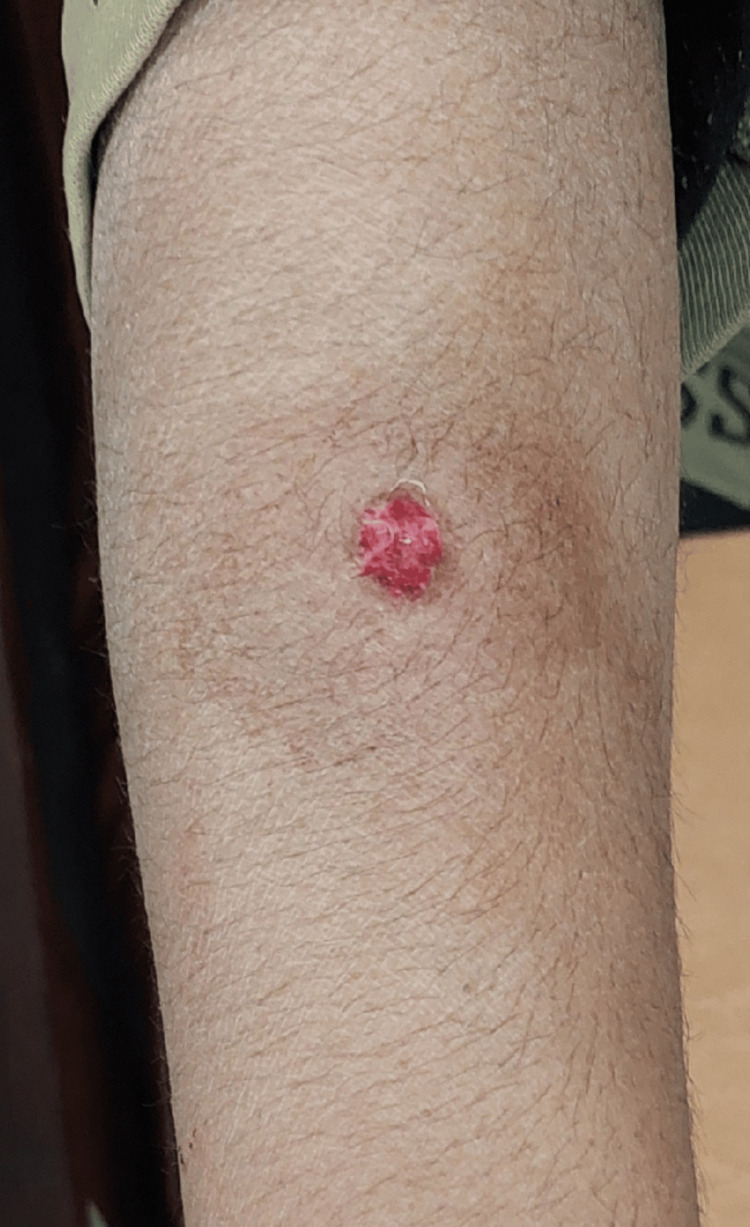
Entry wound on the dorsum of the right forearm.

On physical examination, he was alert and conscious, hemodynamically stable, and afebrile, with no other systemic symptoms. A 1.5 cm × 1 cm lesion with clean margins was observed on the dorsal aspect of his right forearm (Figure 1). There was no swelling, inflammation, or any other sign of infection surrounding the wound. Motor and sensory functions were intact in his right arm, with a normal range of motion at the shoulder, elbow, wrist, and fingers. Distal pulses were palpable and symmetrical.

**Figure 2:**
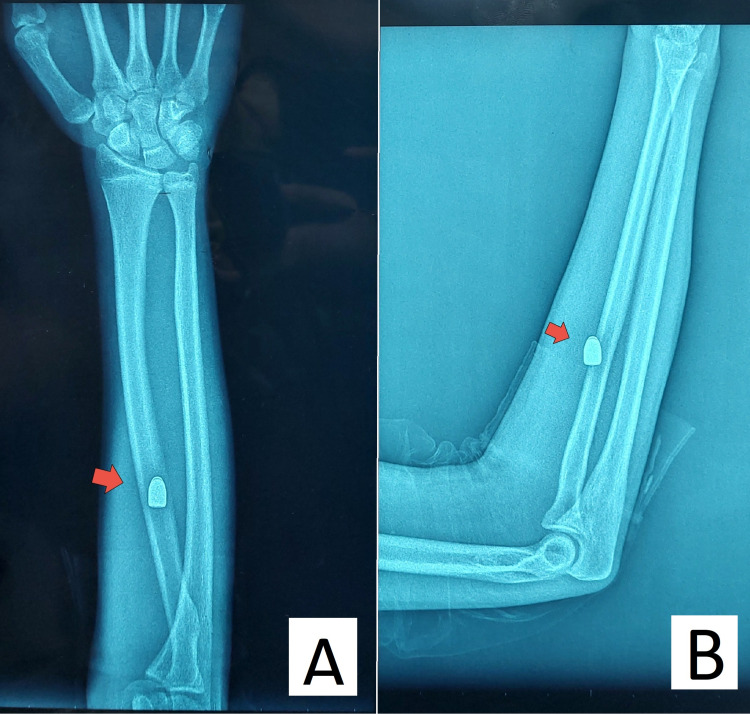
X-ray of the right forearm. A: Anteroposterior view of the right forearm. The red arrow indicates the bullet located extraperiosteally near the right radius. B: Lateral view of the right forearm. The red arrow indicates the bullet positioned anteromedially to the right radius.

An X-ray (anteroposterior and lateral views) was ordered, which revealed a well-defined radiopaque foreign body, consistent with a bullet, embedded in the soft tissue between the distal radius and ulna (Figure 2). No bony injury, fracture, or foreign body fragmentation was observed, consistent with the physical examination findings. The bullet appeared to be located extraperiosteally, without impingement on adjacent structures.

The findings were then shared with the patient’s guardian, who was surprised by the diagnosis. They explained that they had initially considered not coming to the hospital, as the injury did not seem serious. They were counseled on potential complications, including infection, lead toxicity, and possible future issues with movement or pain. Following a surgical consultation and with the guardian’s consent, a decision was made to proceed with surgical removal of the bullet.

The wound was thoroughly irrigated and dressed, and the patient was administered tetanus prophylaxis. He was admitted to the surgical ward for further management. Initial laboratory investigations, including complete blood count, renal function tests (RFTs), liver function tests (LFTs), clotting profile, ESR, and CRP, were all within normal limits. He subsequently underwent pre-anesthetic clearance for surgery the next day, which included a chest X-ray, airway assessment, and cardiopulmonary examination. He was classified as ASA I and cleared for surgery.

**Figure 3:**
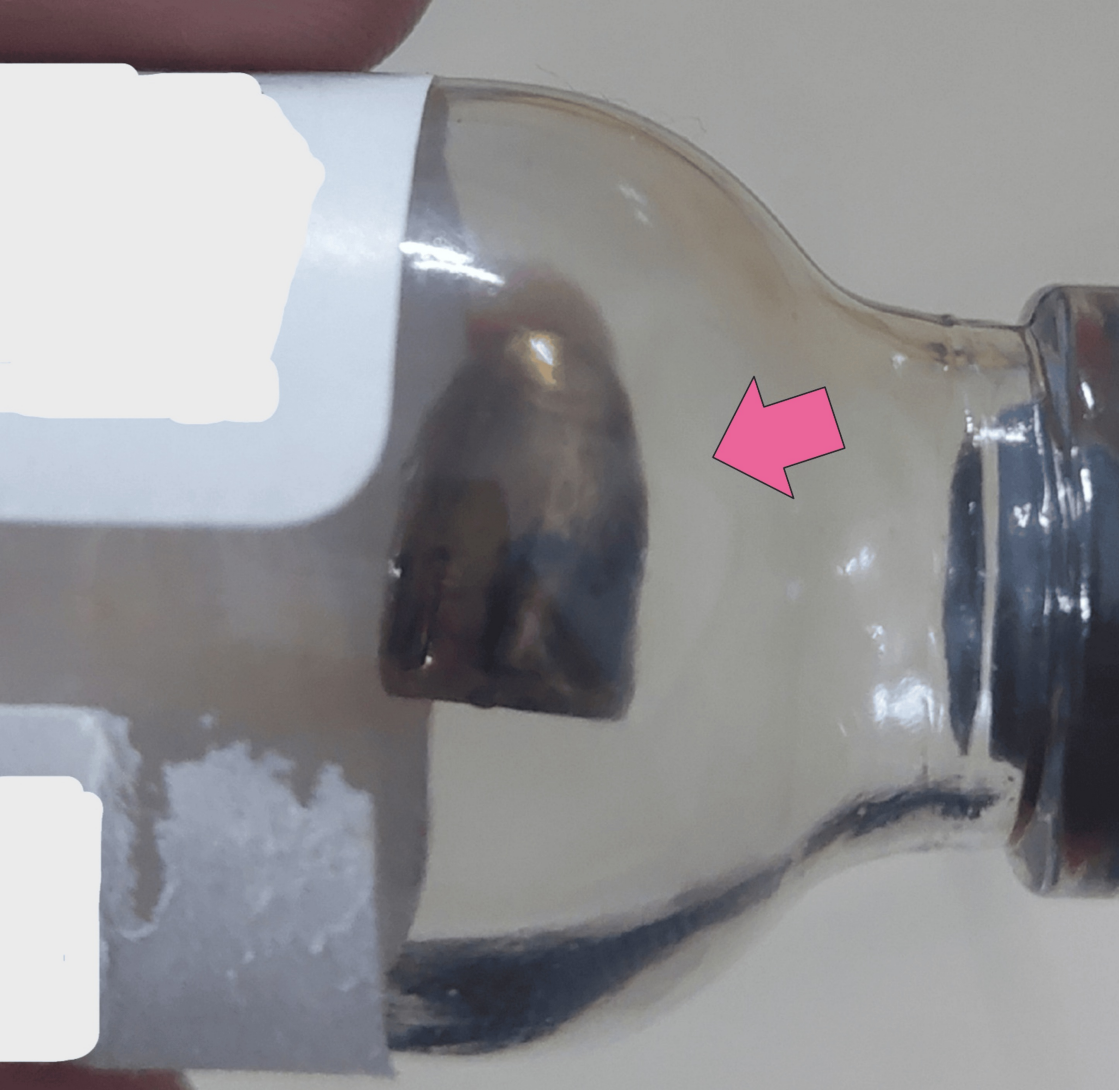
The removed bullet preserved in a recycled glass bottle (red arrow).

The bullet was successfully removed the following day under sterile conditions. A single preoperative dose of 1 g ceftriaxone IV was administered as antibiotic prophylaxis. Intraoperatively, after general anesthesia was administered, a 3 cm incision was made on the dorsal forearm, marked according to the bullet’s position as seen on the X-ray. The bullet was located in the subcutaneous soft tissue, with no involvement of neurovascular structures and no injury to the muscles. It was successfully removed without complications, and the incision was closed using Prolene suture. The bullet was preserved in a container for forensic purposes, if required (Figure 3).

The patient had an uneventful postoperative recovery and was discharged 24 hours after surgery. A follow-up visit after two weeks was advised for wound examination and suture removal.

The unexpected finding in this case emphasizes the importance of performing basic imaging, especially when the cause of injury is unknown. Furthermore, as in this case, patients may present asymptomatically; however, a basic diagnostic workup must still be conducted.

## Discussion

Unintentional firearm injuries are on the rise worldwide, especially in countries where aerial firing is a culturally ingrained practice. In such countries, such as Pakistan, celebratory aerial firing is common during festive events, including marriage ceremonies, New Year’s celebrations, Pakistan Independence Day, and even after national sports team victories [2,4]. GSWs, including those from stray bullets, have serious primary outcomes, and most non-fatal cases also lead to long-term complications and psychological trauma [2,9].

In unique cases, the geographical setting also holds great significance for diagnosis, as similar presentations with unusual signs and symptoms may be recognized. For example, a previous case involving the incidental finding of a bullet in a child’s head resulting from aerial firing was reported in the same hospital [5].

Unlike conventional GSWs sustained at close range, those caused by falling or ricocheted bullets often present with atypical morphology. Entry wounds are small, round, and clean-edged, lacking the surrounding abrasions or soot deposition associated with high-velocity injuries, and exit wounds are frequently absent [10]. This subtle presentation can lead to delays in hospital presentation and diagnosis, increasing the risk of morbidity and mortality [11].

Imaging techniques play a vital role in diagnosing unexplained injuries to the extremities. The management protocol for assessing patients is based primarily on hemodynamic status, along with a thorough physical examination. Plain radiography is preferred for hemodynamically stable patients to identify ballistic fragments. Adjuvant CT with IV contrast is indicated only when prominent signs of vascular injury are found during clinical assessment in hemodynamically stable patients. Furthermore, CT and CT angiography are the preferred modalities for determining the ballistic trajectory and identifying the anatomical structures at risk [12].

Beyond the initial presentation, it is crucial to recognize the long-term complications of firearm injuries, especially those involving retained bullets. Numerous cases have reported delayed complications of retained bullets, including chronic pain, infection, impaired mobility, and bullet migration, which may eventually necessitate removal of the foreign body [1,7]. One study showed that 13 of 116 (11.2%) patients discharged with a retained fragment later developed related complications, with four requiring removal. The study also found that the most common sites for complications were the upper and lower extremities (76.9%) [1]. Another significant but often underdiagnosed complication of retained bullets is lead toxicity (plumbism), which presents with subtle signs and symptoms but can have serious outcomes [8]. Therefore, the long-term complications of retained bullets must be a key consideration when determining management strategies for firearm injuries.

Management of retained bullet fragments is influenced by several factors, including anatomical location, associated symptoms, potential complications, and patient preference [1,7]. In a survey of 427 members of the Eastern Association for the Surgery of Trauma (EAST), Smith RN et al. reported that only 14.5% of respondents’ institutions had a formal policy for retained bullet removal. The most frequently cited indications for removal were pain (88.1%), palpable fragments (71.2%), and infection (40%) [13]. Although formal guidelines are lacking, available evidence supports risk stratification to guide management and removal decisions. High-risk situations include bullets retained within vascular or intra-articular spaces, those causing pain or neurovascular compression, restricted motion, palpable fragments, or elevated blood lead levels (BLL). In such cases, prophylactic removal is generally recommended [7,8,14]. For asymptomatic cases, an individualized approach is advised when considering surgical removal. Additional factors, such as the patient’s ability to attend follow-up visits, access to BLL monitoring, and the risk of postoperative complications, should also be considered.

## Conclusions

Firearm injuries are more common in certain geographical and socioeconomic settings. In such regions, stable patients presenting with unexplained small wounds should also be evaluated for possible firearm injury, as they may be unaware of the cause. Imaging in these cases can be highly useful: X-rays can reveal retained fragments and gross tissue or bone damage, while CT scans with or without IV contrast can provide additional information on the bullet’s trajectory, localization of fragments, and the extent of structural damage. Although multiple complications can arise from a retained metallic foreign body, the surgeon must carefully weigh the risks and benefits of an interventional approach and make an informed decision with the patient or guardian regarding whether to surgically remove the fragments or adopt a wait-and-watch approach.
